# Heterobifunctional PEG Ligands for Bioconjugation Reactions on Iron Oxide Nanoparticles

**DOI:** 10.1371/journal.pone.0109475

**Published:** 2014-10-02

**Authors:** Maarten Bloemen, Thomas Van Stappen, Pieter Willot, Jeroen Lammertyn, Guy Koeckelberghs, Nick Geukens, Ann Gils, Thierry Verbiest

**Affiliations:** 1 Department of Chemistry, KU Leuven, Heverlee, Leuven, Belgium; 2 Department of Pharmaceutical and Pharmacological Sciences, KU Leuven, O&N II, Leuven, Belgium; 3 Department of Chemistry, KU Leuven, Heverlee, Leuven, Belgium; 4 BIOSYST-MeBioS, KU Leuven, Heverlee, Leuven, Belgium; 5 PharmAbs, The KU Leuven Antibody Center, KU Leuven, O&N II, Leuven, Belgium; University of Helsinki, Finland

## Abstract

Ever since iron oxide nanoparticles have been recognized as promising scaffolds for biomedical applications, their surface functionalization has become even more important. We report the synthesis of a novel polyethylene glycol-based ligand that combines multiple advantageous properties for these applications. The ligand is covalently bound to the surface via a siloxane group, while its polyethylene glycol backbone significantly improves the colloidal stability of the particle in complex environments. End-capping the molecule with a carboxylic acid introduces a variety of coupling chemistry possibilities. In this study an antibody targeting plasminogen activator inhibitor-1 was coupled to the surface and its presence and binding activity was assessed by enzyme-linked immunosorbent assay and surface plasmon resonance experiments. The results indicate that the ligand has high potential towards biomedical applications where colloidal stability and advanced functionality is crucial.

## Introduction

The potential of iron oxide nanoparticles (NP) in biomedical applications is widely recognized: they can act as magnetic resonance imaging (MRI) contrast agents, superparamagnetic carriers for drugs or are used in hyperthermia treatments. [1]–[6] By improving the synthesis of these particles, their quality and availability has largely increased. [7]–[12] When NP are used in biomedical applications, two requirements are often necessary. First, their colloidal stability in complex environments is crucial. If the particles become unstable in for instance blood, they will precipitate, possibly triggering severe inflammatory responses. [13]–[15] Secondly, they should possess accessible anchor points for molecules or proteins to be coupled onto. This allows NP to selectively interact with certain targets or to carry drugs close to a desired location.

However, functionalization of their surface has proven to be non-trivial. Although multiple different approaches have been developed, most of them lack a certain degree of control. [16] Coating their surface with functional polymers is a straightforward method, but has crosslinking issues and allows little control over the thickness of the layer and orientation of functional groups. [17] Since they are not covalently attached to the surface, they could potentially detach, which would make the particles precipitate. Growing an additional silica layer on the iron oxide core, on the other hand, has several advantages: the shell thickness can be well controlled and it is chemically inert. [18] However, the diameter of such NP increases by several nanometers, which is often not desired for biomedical applications. [19] This problem was circumvented by the introduction of functional siloxane molecules on iron oxide NP. They also form a silicon dioxide shell, albeit very thin, and they contain a functional group, which can have several advantages or uses later on. [20], [21].

Even though multiple variants of these silanes are commercially available, they often do not have the desired structure or properties. This can easily be related to the complicated handling of siloxane molecules. Since they react with water and are relatively intolerant to heat, modification reactions have to be limited in time and workup. Tucker-Schwartz *et al.* recently published an easy method to avoid this direct modification of the siloxanes, by adopting thiol-ene click chemistry. [22] Their approach allows to synthesize a very complex molecule first and attach a siloxane group as the final step. Click chemistry is a concept rather than a specific reaction, which comprises fast reactions with very high yields and non-aggressive by-products. [23], [24] In addition the reaction should be modular and have relatively simple reaction conditions. Very well-known examples are copper mediated azide-alkyne cycloadditions, thiol-ene and Diels-Alder reactions. [24], [25] In this manuscript we developed a new ligand, based on a polyethylene glycol (PEG) backbone, and transformed it into a siloxane by straightforward thiol-ene click chemistry. By modifying the end-group of the backbone, functional groups were easily introduced onto the nanoparticle’s surface. The high purity and straightforward synthesis of the ligand makes this method very valuable for large scale and reproducible functionalization of iron oxide nanoparticles. This universal method requires only basic knowledge of organic chemistry and can be widely applicable by scientists without a substantial chemistry background.

To investigate the full potential of the ligand, several antibodies (Ab) were coupled to its anchor groups (carboxylic acids) and their activity was assessed via fiber optic surface plasmon resonance experiments. As a model system, an antibody (MA-33H1F7) targeting the serpin plasminogen activator inhibitor-1 (PAI-1) protein was selected. [26] This protein is an important factor in the plasminogen-plasmin system since it inhibits plasminogen activators tissue-type plasminogen activator and urokinase, which are involved in clot formation and degradation processes in blood. [27] These Ab were coupled to the NP by using popular EDC-NHS (1-ethyl-3-(3-dimethylaminopropyl)carbodiimide hydrochloride, *N*-hydroxysuccinimide) chemistry and their presence was investigated by ELISA (enzyme-linked immunosorbent assay). To assess their potential in biomedical applications, their colloidal stability was tested in undiluted human plasma and serum. The results indicate that the developed ligand has high potential because of its elegant synthesis, its positive influence on the colloidal stability of the nanoparticle as well as its properties for antibody coupling chemistry.

## Experimental

### 1. Materials

2,2-dimethoxy-2-phenylacetophenone (DMPAP, 99%), 4-dimethylaminopyridine (DMAP, >99%), succinic anhydride (>99%), 1-Ethyl-3-(3-dimethylaminopropyl)carbodiimide (EDC) and mercaptopropyltrimethoxysilane (95%) were purchased from Sigma Aldrich. Allyl-PEG10-OH was obtained from Polysciences, Inc. Triethylamine was ordered at Janssen Chimica. N-hydroxy succinimide (98+%) was obtained from Alfa Aesar. 2-(N-morpholino)ethanesulfonic acid monohydrate (MES) was purchased at Fluka. The monoclonal antibodies (host: mouse) used in this study are MA-33H1F7 (target: human PAI-1/t-PA complex) and MA-T12D11 (target: human TAFI), supplied by the Therapeutic and Diagnostic Antibodies group of the KU Leuven. [26].

All ultrasonication steps were performed in a Branson 5510 sonicator bath. Fourier transform infrared spectra were measured using a Bruker Alpha FT-IR spectrometer equipped with a Platinum ATR module.

### 2. Carboxylic acid-terminated PEG

In a 50 ml flask, allyl-PEG10-OH (1eq, 4,00 mmol, 1.992 g) was mixed with succinic anhydride (1.1eq, 4.40 mmol, 440 mg) and 4-dimethylaminopyridine (DMAP) (0.02eq, 0.08 mmol, 9.7 mg). This mixture was stirred and heated to 50°C for 16 hours. The resulting product was purified twice by precipitation in cold diethyl ether, centrifugation and drying in vacuum. ^1^H NMR (300 MHz, CDCl_3_): δ (ppm) 2.65 (s, 4H), 3.6–3.7 (m, 38H), 4.02 (d, 2H), 4.26 (t, 2H), 5.16–5.30 (m, 2H), 5.8–6.0 (m, 1H). ^13^C NMR (75 MHz, CDCl_3_): δ (ppm) 29.2, 29.5, 63.8, 68.9, 69.3, 70.5, 72.2, 117.1, 134.7, 172.1. MS (chemical ionization, isobutane): m/z = 499 (ester fragment, M^+^ - C_4_O_3_H), 101 (ester fragment, M^+^ - C_23_O_11_H_46_).

### 3. Thiol-ene click chemistry

To form the siloxane-terminated PEG molecule, allyl-terminated PEG (mixture of modified and unmodified, 1 mmol) was mixed with (3-mercaptopropyl) trimethoxysilane (1eq, 1 mmol, 185.7 µL) and 2,2-dimethoxy-2-phenylacetophenone (DMPAP, 0.05eq, 0.05 mmol, 12.8 mg). This mixture was stirred for 1 hour in a UV chamber, equipped with 3 LEDs (365 nm, output power 200 mW). If smaller quantities are used, a small amount of chloroform can be added to improve the stirring. [22] The product was used without further purification. ^1^H NMR (300 MHz, CDCl_3_): δ (ppm) 0.76 (t, 2H), 1.70 (m, 2H), 1.85(m, 2H), 2.55 (m, 4H), 2.64 (s, 4H), 3.57 (s, 9H), 3.5–3.8 (m, 40H), 4.26 (t, 2H).

### 4. Nanoparticle functionalization

The synthesis of iron oxide NP as well as the introduction of siloxanes onto their surface was performed as reported in our previous manuscript. [20] In general, 1 mmol of siloxanes is mixed with 100 mg of Fe_3_O_4_ NP in 50 mL of toluene. To this mixture 2.5 mL of triethylamine and 50 µL of water are added. The solution was placed in a ultrasonication bath for 5 hours, after which 50 mL of heptane was added to precipitate the particles. Afterwards, they were attracted magnetically and washed 3 times with acetone. Finally the particles were dried in vacuum and dispersed in MilliQ water (with a concentration up to 20 mg/mL).

### 5. Protein coupling

The concentrated nanoparticle solution was diluted in 50 mM 2-(N-morpholino)ethanesulfonic acid (MES) buffer, pH 5.5, to reach a final concentration of 3 mg/mL. 0.75 mg 1-Ethyl-3-(3-dimethylaminopropyl)carbodiimide (EDC) and 0.75 mg N-hydroxysuccinimide (NHS) was added to 1 ml of this solution and shaken for 20 minutes to activate the carboxylic acids. The antibodies were diluted in 2 mL of the same MES buffer after which both solutions were mixed and shaken for 1 hour. To separate the particles from the solution, a Miltenyi Biotech MS magnetic column was used. After rinsing the column with MilliQ water, the nanoparticle dispersion was run through the column, which was placed inside a circular magnet. The column was washed 2 times with 1 mL of sodium phosphate buffer (20 mM, pH 7). To elute the particles, the column was removed from the magnet and 1 mL of phosphate buffer was used as eluent.

### 6. ELISA

In the ELISA assay, recombinant plasminogen activator inhibitor (PAI-1) is coated on the plate and free binding sites are blocked with bovine serum albumin. Samples are applied in different dilutions as well as a standard curve of MA-33H1F7. [26] After incubation, horseradish peroxidase (HRP) conjugated rabbit anti-mouse IgG (Sanbio B.V., Uden, The Netherlands) is applied, followed by an o-phenylenediamine (OPD) induced colorimetric reaction. The intensity of the color is directly correlated with the amount of bound MA-33H1F7. Sample values are calculated using the standard curve.

### 7. Surface plasmon resonance (SPR)

An optical fiber was first coated with a gold layer, which was subsequently covered with a self-assembling monolayer (SAM). This thiol- and carboxyl-terminated molecule was obtained from Dojindo molecular technologies. The SAM was activated by a solution containing 0.4M EDC and 0.1M NHS in a 50 mM MES buffer (pH 6.0) for 20 minutes. Afterwards the fiber was brought into a solution containing the antigen, PAI-1 (24 µg/mL) for 25 minutes. Finally the fiber was transferred into a blocking solution (0,1% tween and 0.05% BSA). All subsequent experiments were performed with 1 mg/ml nanoparticle solutions.

## Results and Discussion

**Figure 1:**
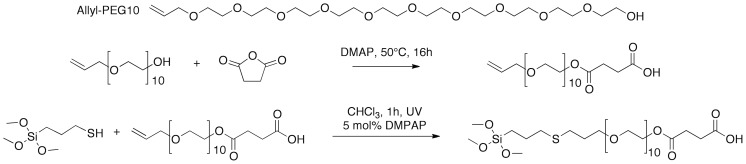
Reaction scheme. Allyl-terminated polyethylene glycol was modified by reaction with succinic anhydride. 4-dimethylaminopyridine (DMAP) catalyzes this reaction. Subsequently the allyl functionality is reacted with a thiol-containing siloxane molecule, by thiol-ene click chemistry, which yields the final carboxylic acid-terminated PEG-siloxane.

The novel PEG-siloxane ligand was designed bearing two important characteristics in mind: having one accessible functional group and providing excellent colloidal stability to the nanoparticle. To ensure the first property, a PEG oligomer end-capped with an allyl functionality was modified with succinic anhydride. This reaction was performed without solvent, since the anhydride dissolves in PEG at elevated temperatures. 4-Dimethylaminopyridine (DMAP) was added, as a catalyst, to speed up the reaction. [28] The available hydroxyl group at the end of the PEG chain reacts with the anhydride, resulting in a free carboxylic acid (see Figure 1). This product was purified once by precipitating it in diethyl ether, which removed traces of the catalyst and excess anhydride.

The second step of the ligand synthesis involved a click chemistry reaction. We opted for this approach, since working with siloxanes is difficult. They react with moisture and are not resistant to prolonged heating. [22] The thiol-ene click chemistry, on the other hand, is fast and takes place at room temperature. Another great advantage of this approach is that the final siloxane molecule can be added directly to the functionalization solution, without additional workup. Any traces of the radical initiator or its by-products are inert in this reaction.

**Figure 2:**
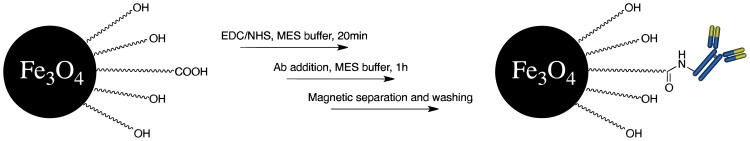
Bioconjugation strategy. The available carboxylic acid groups are activated with EDC-NHS chemistry. The resulting NHS ester reacts with amine groups of the antibody in a MES buffer. Finally the particles are recovered from the supernatant by a magnetic column.

Even though the functionalized PEG molecule could provide sufficient steric hindrance, which ensures colloidal stability of the nanoparticle, we chose to mix modified and unmodified PEG siloxanes during the functionalization step. [20] Thus, the nanoparticle is covered with a complete PEG shell, where the modified chains are sterically available, since they are longer. From the FTIR data (data in SI, Figures S1 and S2) was derived, that even though the chain length of the ligand is sufficiently short to enhance the stacking of the molecules (crystalline domains), a small percentage is coiled (amorphous domains). [20] Because only a small part of the ligands have a carboxylic acid functionality, the overall pH sensitivity is reduced. The end result (idealized) is shown in Figure 2: carboxylic acid groups are now available as anchor points for future reactions. By Fourier transform infrared measurements, the presence of modified PEG chains was observed (data in SI, Figures S1 and S2). All further experiments were conducted on nanoparticles coated with 90% unmodified and 10% modified PEG siloxanes (molar percentages).

These functionalized nanoparticles show excellent colloidal stability in multiple different environments. Similarly to our previous report, we tested the stability in undiluted human serum and plasma (data in SI, Figure S3). [20] The nanoparticles (8.6±0.6 nm, Transmission Electron Microscopy data in SI, Figure S4), coated with mixed siloxanes, clearly showed the properties of both PEG and carboxylic acids. In particular they show enhanced stability in pH ranges above 5, where the carboxylic acids are charged (picture in SI, Figure S5). In media like serum or plasma, no precipitation was observed, even after 25 hours at room temperature without agitation. NP were also coated with 100% modified PEG siloxanes, but these particles had significantly lower colloidal stability in these acidic environments (pH 5–6), due to the lack of stabilizing charges. Steric stabilization by the PEG chains was not sufficient in this case, since to much carboxylic acids were present. We therefore decided to focus on nanoparticles with mixed siloxane coatings.

Covalent attachment of the selected Ab onto the NP was performed via a standard EDC-NHS coupling. [29] The mechanism is based on the activation of the carboxylic acid with EDC, which forms an unstable acylisourea intermediate. This intermediate reacts with NHS to form a stable ester that exhibits enhanced stability in aqueous environments. Although this extra step is not strictly necessary, it greatly improves the binding efficiency, by reducing the occurrence of side reactions on the acylisourea intermediate. All reactions were performed in a slightly acidic buffer (MES 50 mM, pH5.5), which improves the final coupling reaction on two domains. First, the low pH enhances the activation of the carboxylic acid by EDC. [30] Secondly, the formed NHS ester has substantially lower hydrolysis rates below pH7. [30] Further protein crosslinking (second, third, … layer) is reduced by the slow reaction rate of the partially protonated amines. [30] A slower reaction rate was preferred in this procedure, since the formation of a protein corona is also a thermodynamically favorable process. [31] A higher reaction rate could result in coating the NP with multiple layers of proteins and crosslinking between different NP. Afterwards the conjugated NP were purified by a magnetic column, which has a very large surface area, since normal attraction with a magnet was too time-consuming. This was necessary because of the excellent colloidal stability of the NP in the buffer, which dramatically slows down the attraction rate. If the NP were precipitated by a highly concentrated salt solution, it was difficult to redisperse them afterwards. Using a magnetic column also enabled us to wash the particles while they were retained on the column. After removing the magnetic field from the column, the particles were easily collected by eluting with a PBS buffer.

In this study, we opted for two different Ab: MA-33H1F7, targeting PAI-1, and MA-T12D11, targeting TAFI, as the negative control. [26], [32].

**Figure 3:**
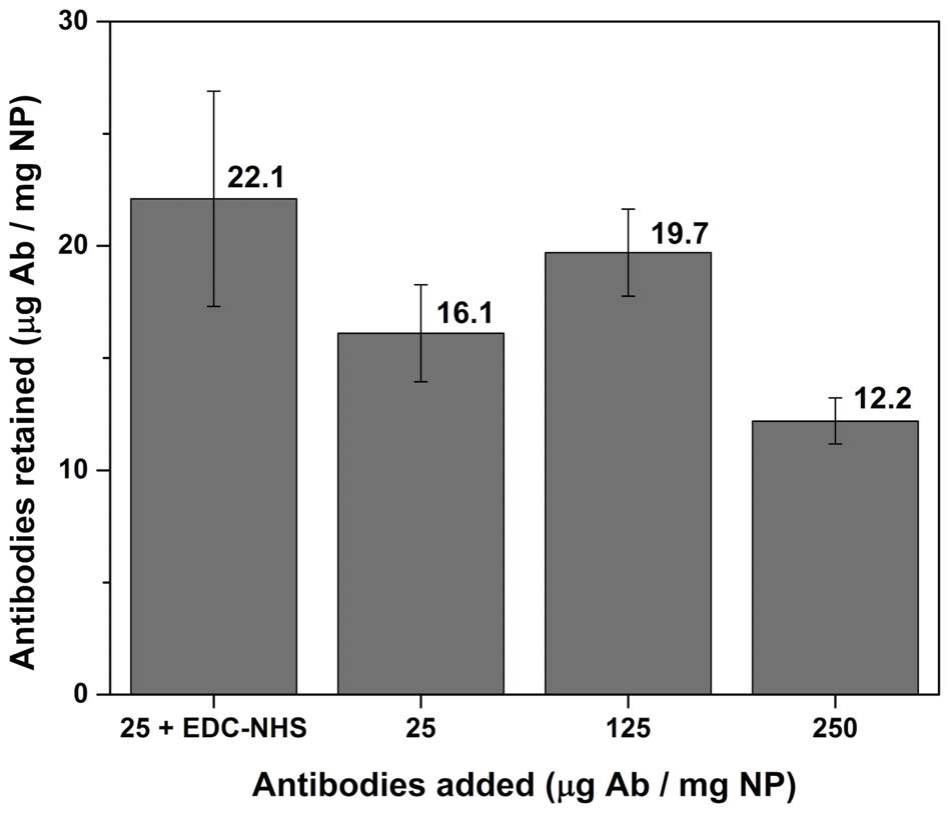
Nanoparticle-antibody coupling results. If EDC-NHS coupling reagents are added to the mixture of NP and antibodies, slightly more proteins are retained on the NP. This indicates that a small level of crosslinking occurs. When a large amount of antibodies (without coupling reagents) is added, no significant difference is observed, which shows that only a hard corona remains on the NP after washing. All error bars are shown as the percentage error on the total value.

Multiple methods are available to determine the concentration of proteins on NP; however not all are appropriate when iron oxide is involved. Colorimetric methods like the Bradford assay are influenced by the strong light absorption of the black NP, which makes the results difficult to interpret. [33] Fourier transform infrared spectroscopy can only confirm the presence of proteins but is not appropriate for assessing the concentration. We opted for an ELISA assay in this case, whereby the remaining proteins in the supernatant and washing fractions were determined. This way, the amount of proteins on the surface of the NP can easily be calculated. As a comparison, NP and Ab were also mixed in the absence of coupling reagents, so only aspecific adsorption could occur (protein corona formation). [31], [34] Hence this would set a benchmark for the protein concentration of the hard corona formation (without possible protein crosslinking). [35], [36] When the concentration of proteins was increased ten times (see Figure 3), the amount of adsorbed proteins does not change significantly. This indicates that the washing steps remove all proteins, except the hard corona, which is more strongly attached to the surface. [35]–[37] Since all three experiments without coupling reagents (including error bars) give a similar value, we learned that the hard protein corona corresponds to 15–20 micrograms of proteins per milligram nanoparticles, which is similar to literature for particles of comparable size and shape. [38]–[43] When we added the coupling reagents (25+ EDC-NHS), we obtained a result that was comparable, but slightly higher in value. This indicates that a small amount of crosslinking is occurring, which can be expected for EDC-NHS reactions involving proteins. However the coating of the nanoparticles is close to the optimal value (solely the hard corona), which underlines the quality of the coating and the coupling procedure.

**Figure 4:**
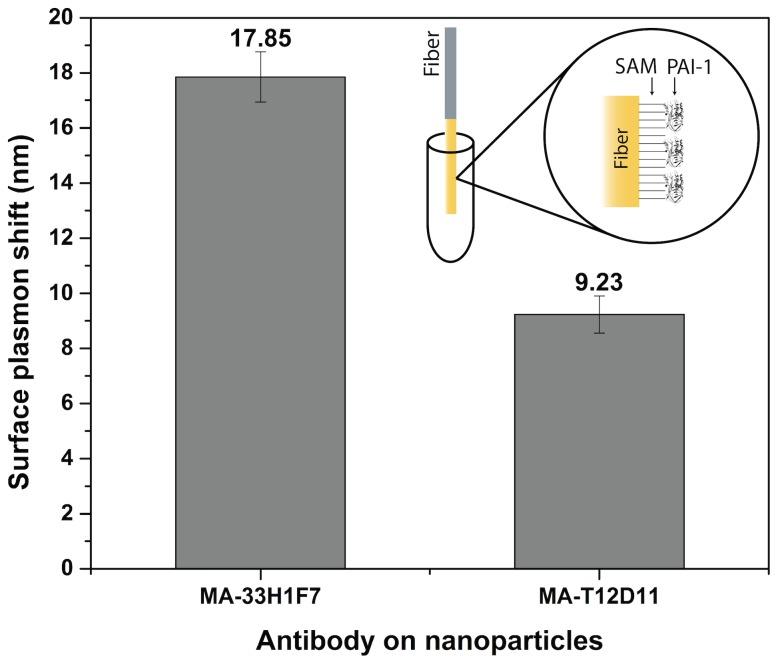
SPR fiber results. A multimode optical fiber was coated with a gold layer, a self-assembling monolayer (SAM) and the appropriate antigen (PAI-1), as shown in the inset. The SPR shift caused by the nanoparticles, coated with MA-33H1F7 or MA-T12D11, is clearly visible.

Although an ELISA assay can determine the loading capacity, it is incapable of assessing the activity of the coupled antibodies on the spherical nanoparticles. To investigate the ability of the Ab to recognize their antigen (Ag), the nanoconjugates were brought into contact with a PAI-1-coated surface plasmon resonance (SPR) optical fiber. By looking at the shift in the plasmon wavelength, the interaction between Ab and Ag can be assessed. A standard multimode optical fiber was coated with a gold layer and a self-assembling monolayer with carboxylic acid end-groups. [44] To these groups, PAI-1 was coupled via EDC-NHS chemistry (Figure 4). When the nanoparticles were brought into contact with the fiber, they induced a shift in the plasmon resonance relative to their binding efficiency. The binding, as a whole, is the sum of two separate interactions: the protein corona effect and the antibody-antigen (Ab-Ag) bond. The first is caused by the aspecific interaction between the Ab and the Ag, similar to the formation of a second (soft) corona, this cannot be avoided and hence is viewed as a background in the signal. The latter, however, is specific for each Ab-Ag couple.

In this experiment, one can clearly see the difference in SPR shift, caused by the Ab-Ag interaction. An extra shift of more than 8 nm was measured by the SPR-fiber setup when comparing the NP, coated with MA-33H1F7 (targeting PAI-1) or MA-T12D11 (targeting TAFI). This result ensures that, although the Ab are coupled in a random fashion, their activity is retained and they are still partially sterically accessible. We hypothesize that a large fraction of the Ab indeed lose their activity due to an unfavorable direction of bonding. However, the strongly curved, large surface of the NP allows a high overall antibody loading capacity that partially compensates for the losses in activity. Future experiments will focus on employing a more directional coupling strategy, which will give us more insight in this complex relation.

The excellent colloidal stability of the NP, coated with the PEG-ligand, will allow to use the particles for various biomedical applications. Since the ratio of functional ligands can easily be adjusted, a library of mixed-monolayer nanoparticles can be synthesized for future experiments. Similarly, the core size of the NP can be varied, to control the overall size of the bioconjugates. This can have an important influence on their cell uptake or retention time in vivo. [45], [46] Moreover, they can serve as a platform for the bioconjugation of proteins for multiple applications like selective magnetic separation or MRI contrast agents.

## Conclusions

In order to fully customize the surface coating of iron oxide nanoparticles, a PEG building block was modified with carboxylic acid groups and afterwards attached to a siloxane via thiol-ene click chemistry. These ligands were introduced onto the nanoparticles’ surface, which significantly improved the colloidal stability in complex environments. To prove their added functionality, antibodies were coupled to the carboxylic acid end-groups. An ELISA assay was performed to indirectly determine the amount of coupled proteins, while SPR experiments confirmed their activity. Because these ligands provide excellent colloidal stability and can also act as an anchor point for coupling via a simple modification, they have high potential in future nanoparticle design for biomedical applications.

## Supporting Information

Figure S1 **Fourier transform infrared spectrum (FTIR) of the original allyl-PEG10-OH ligand and the modified version.** The ester peak at 1725 cm^−1^ is clearly visible after the ring opening of the anhydride, while the –OH peak around 3500 cm^−1^ disappears.
(DOCX)

Figure S2 **Fourier transform infrared spectrum (FTIR) of the allyl-PEG10-COOH ligand, the oleic acid-coated and the modified iron oxide nanoparticles.** The ester peak is still clearly visible at 1725 cm^−1^, as well as the different polyether vibrations between 1250 and 1500 cm^−1^. The presence of the iron oxide nanoparticles is confirmed by the Fe-O and Si-O vibrations at respectively, 590 and 1100 cm^−1^. The broad peaks at 1660 and 3400 cm^−1^ are due to the presence of water, which remains in the PEG layer.
(DOCX)

Figure S3 **Absorbance of nanoparticle dispersions in plasma and serum.** To verify the stability of the functionalized nanoparticles in complex environments; the absorbance of dispersions in plasma and serum was measured at 1000 nm. The particles were dispersed at 1 mg/mL and the absorbance was monitored for 25 hours. A significant decrease of the absorbance would indicate colloidal instability and precipitation of the nanoparticles.
(DOCX)

Figure S4 **Transmission electron microscopy (TEM) image of the iron oxide nanoparticles (8.6±0.6** **nm).** Their size was determined by ImageJ software.
(DOCX)

Figure S5 **The colloidal stability of the nanoparticle dispersions is excellent, even after 1 year of storage.** The samples (5 mg/mL in water, pH 7) show above have the following coatings (molar percentages): **A**, 100% PEG_10_-OH; **B**, 10% PEG_10_-COOH 90% PEG_10_-OH; **C**, 25% PEG_10_-COOH 75% PEG_10_-OH; **D**, 50% PEG_10_-COOH 50% PEG_10_-OH.
(DOCX)
