# Supplementary material for: Heterobifunctional PEG Ligands for Bioconjugation Reactions on Iron Oxide Nanoparticles
Source: PLoS One. 2014 Oct 2;9(10):e109475. doi: 10.1371/journal.pone.0109475 (PMC4183648; doi:10.1371/journal.pone.0109475)
Supplement: Figure S1 — Fourier transform infrared spectrum (FTIR) of the original allyl-PEG10-OH ligand and the modified version. The ester peak at 1725 cm−1 is clearly visible after the ring opening of the anhydride, while the –OH peak around 3500 cm−1 disappears. (DOCX) [file pone.0109475.s001.docx]

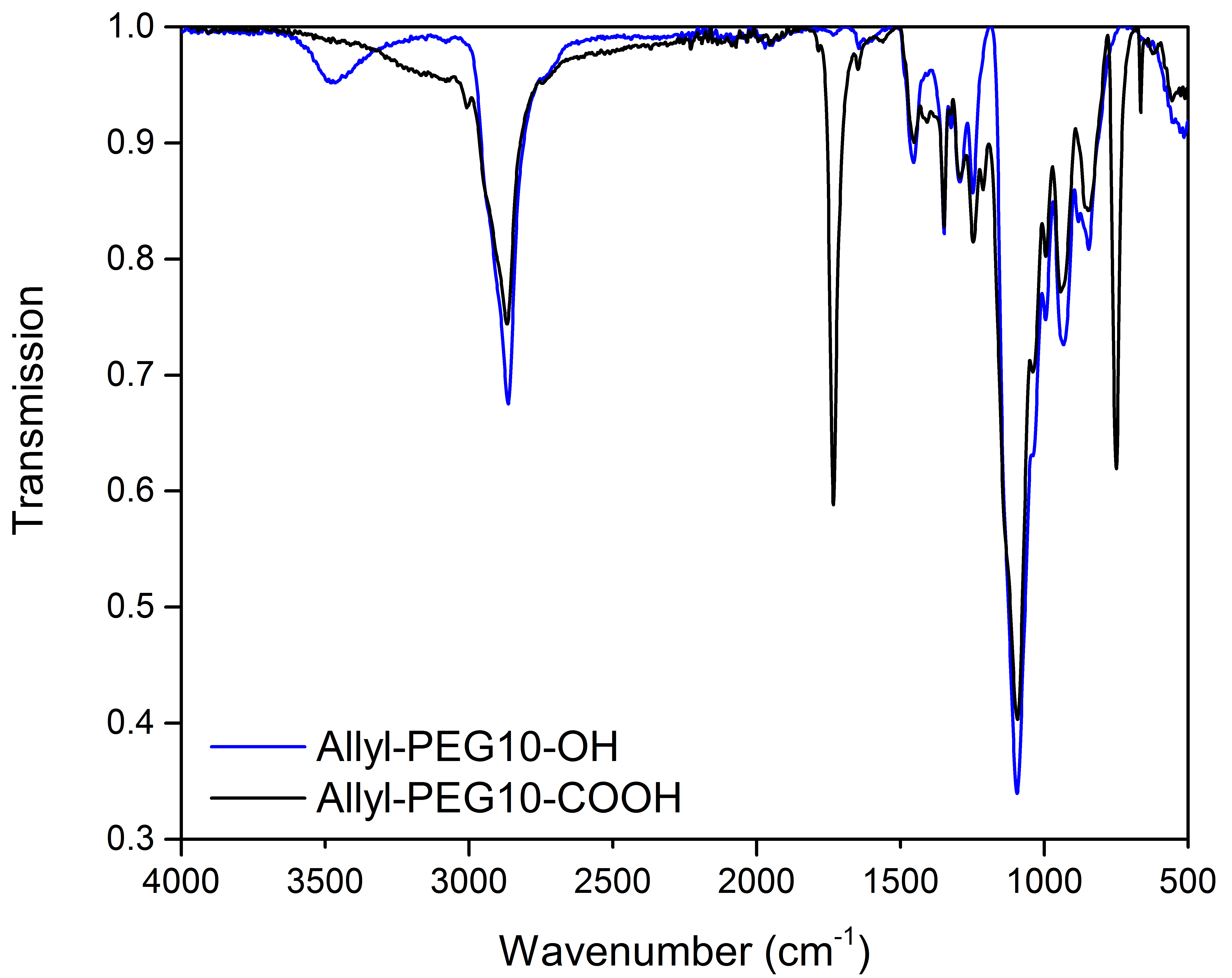


**Figure S1:** **Fourier transform infrared spectrum (FTIR) of the original allyl-PEG10-OH ligand and the modified version.** The ester peak at 1725 cm^-1^ is clearly visible after the ring opening of the anhydride, while the –OH peak around 3500 cm^-1^ disappears.
