# Supplementary material for: Heterobifunctional PEG Ligands for Bioconjugation Reactions on Iron Oxide Nanoparticles
Source: PLoS One. 2014 Oct 2;9(10):e109475. doi: 10.1371/journal.pone.0109475 (PMC4183648; doi:10.1371/journal.pone.0109475)
Supplement: Figure S2 — Fourier transform infrared spectrum (FTIR) of the allyl-PEG10-COOH ligand, the oleic acid-coated and the modified iron oxide nanoparticles. The ester peak is still clearly visible at 1725 cm−1, as well as the different polyether vibrations between 1250 and 1500 cm−1. The presence of the iron oxide nanoparticles is confirmed by the Fe-O and Si-O vibrations at respectively, 590 and 1100 cm−1. The broad peaks at 1660 and 3400 cm−1 are due to the presence of water, which remains in the PEG layer. (DOCX) [file pone.0109475.s002.docx]

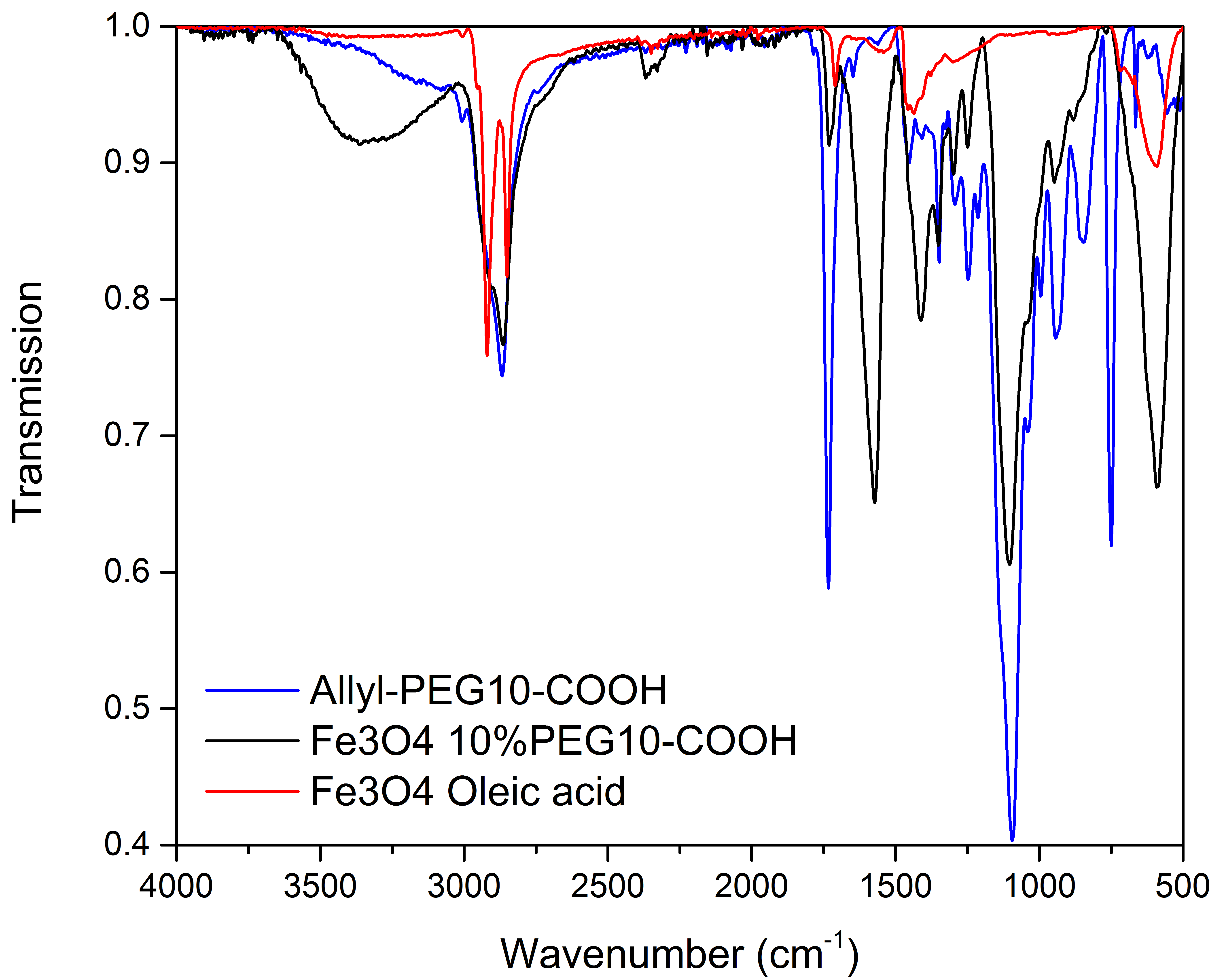


**Figure S2:** **Fourier transform infrared spectrum (FTIR) of the allyl-PEG10-COOH ligand, the oleic acid-coated and the modified iron oxide nanoparticles.** The ester peak is still clearly visible at 1725 cm^-1^, as well as the different polyether vibrations between 1250 and 1500 cm^-1^. The presence of the iron oxide nanoparticles is confirmed by the Fe-O and Si-O vibrations at respectively, 590 and 1100 cm^-1^. The broad peaks at 1660 and 3400 cm^-1^ are due to the presence of water, which remains in the PEG layer.
