# Supplementary material for: Heterobifunctional PEG Ligands for Bioconjugation Reactions on Iron Oxide Nanoparticles
Source: PLoS One. 2014 Oct 2;9(10):e109475. doi: 10.1371/journal.pone.0109475 (PMC4183648; doi:10.1371/journal.pone.0109475)
Supplement: Figure S3 — Absorbance of nanoparticle dispersions in plasma and serum. To verify the stability of the functionalized nanoparticles in complex environments; the absorbance of dispersions in plasma and serum was measured at 1000 nm. The particles were dispersed at 1 mg/mL and the absorbance was monitored for 25 hours. A significant decrease of the absorbance would indicate colloidal instability and precipitation of the nanoparticles. (DOCX) [file pone.0109475.s003.docx]

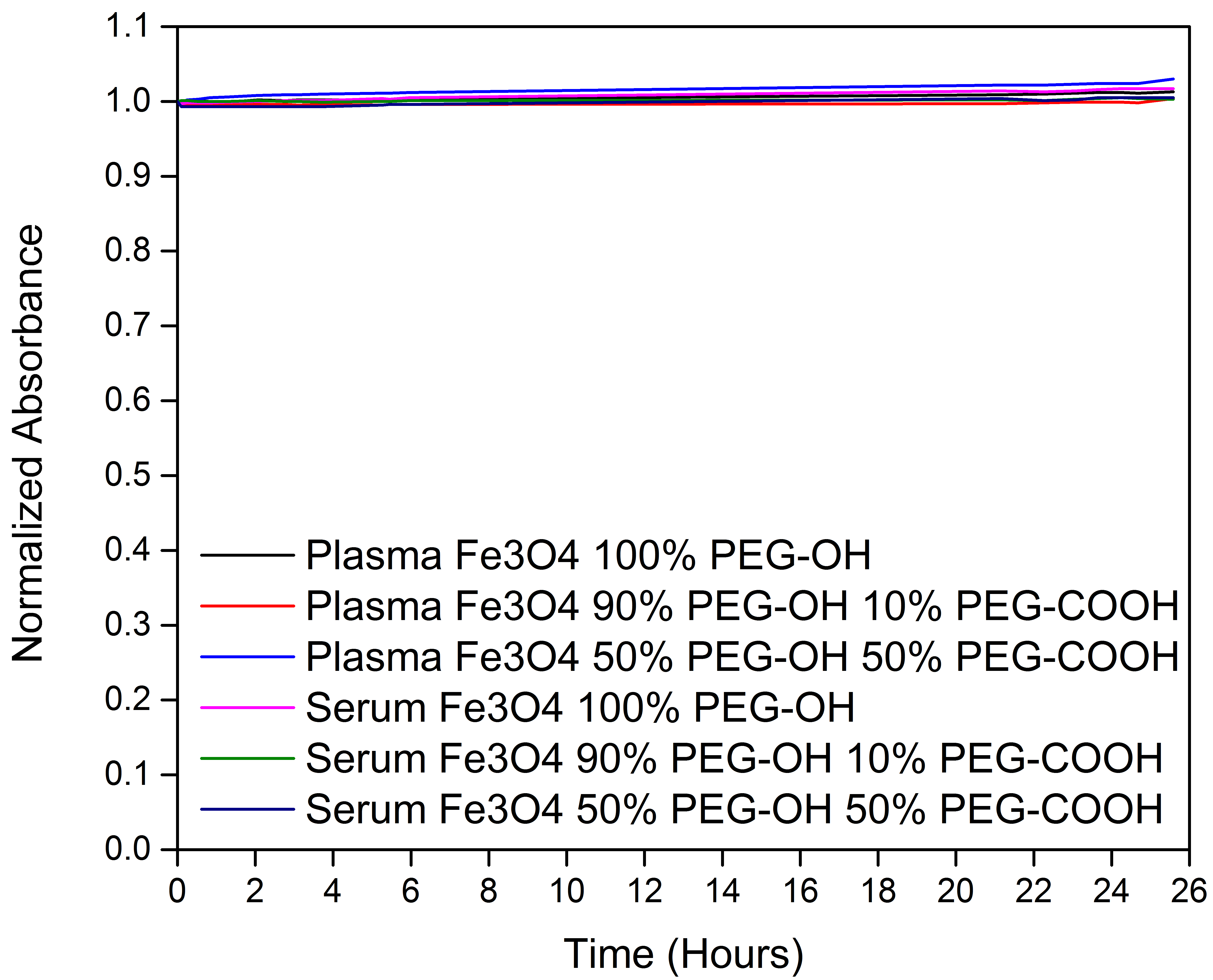


**Figure S3:** **Absorbance of nanoparticle dispersions in plasma and serum.** To verify the stability of the functionalized nanoparticles in complex environments; the absorbance of dispersions in plasma and serum was measured at 1000nm. The particles were dispersed at 1mg/ml and the absorbance was monitored for 25 hours. A significant decrease of the absorbance would indicate colloidal instability and precipitation of the nanoparticles.
