# Supplementary material for: Heterobifunctional PEG Ligands for Bioconjugation Reactions on Iron Oxide Nanoparticles
Source: PLoS One. 2014 Oct 2;9(10):e109475. doi: 10.1371/journal.pone.0109475 (PMC4183648; doi:10.1371/journal.pone.0109475)
Supplement: Figure S4 — Transmission electron microscopy (TEM) image of the iron oxide nanoparticles (8.6±0.6 nm). Their size was determined by ImageJ software. (DOCX) [file pone.0109475.s004.docx]

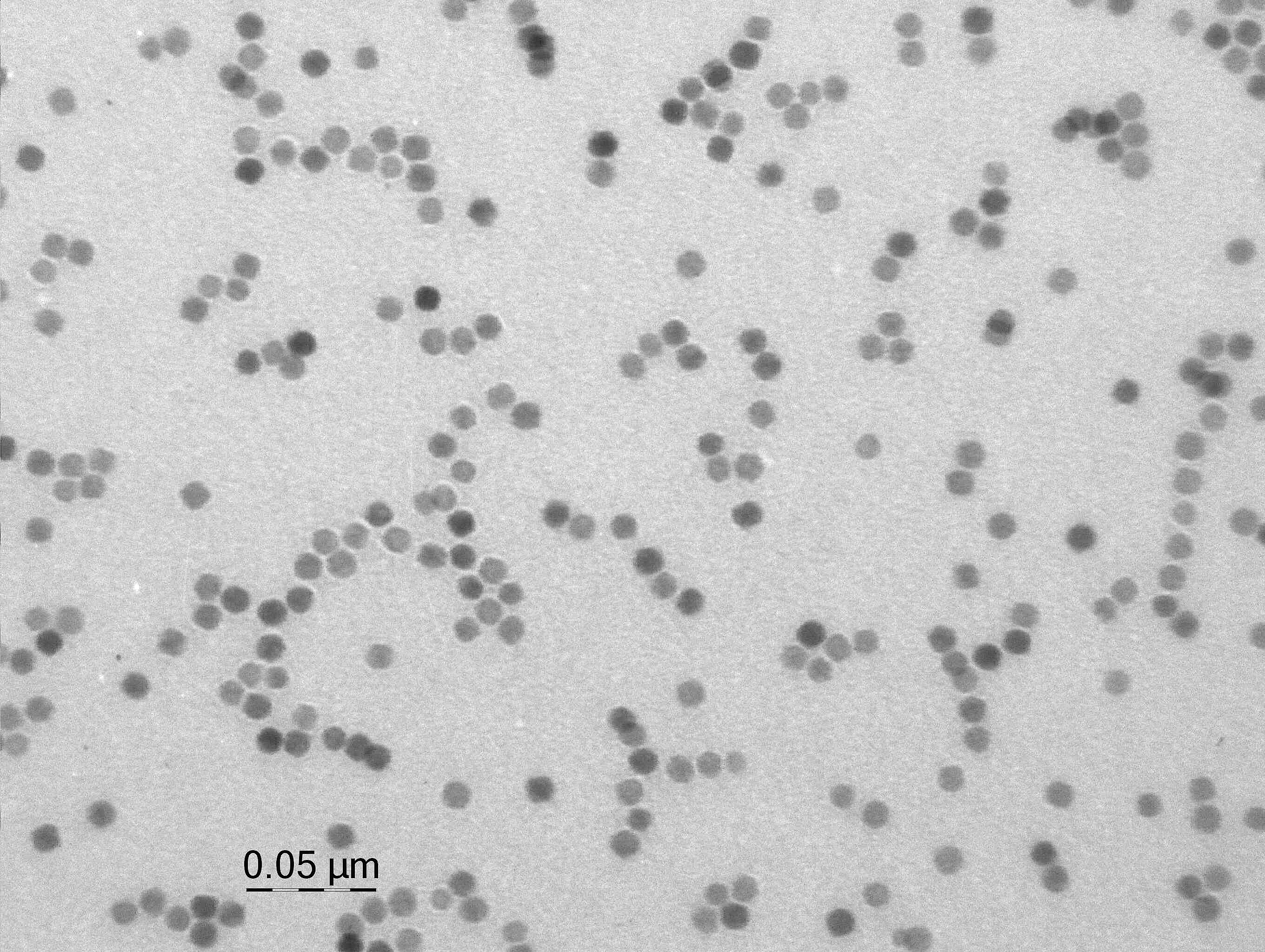


**Figure S4:** **Transmission electron microscopy (TEM) image of the iron oxide nanoparticles (8.6±0.6nm).** Their size was determined by ImageJ software.
