# Supplementary material for: Heterobifunctional PEG Ligands for Bioconjugation Reactions on Iron Oxide Nanoparticles
Source: PLoS One. 2014 Oct 2;9(10):e109475. doi: 10.1371/journal.pone.0109475 (PMC4183648; doi:10.1371/journal.pone.0109475)
Supplement: Figure S5 — The colloidal stability of the nanoparticle dispersions is excellent, even after 1 year of storage. The samples (5 mg/mL in water, pH 7) show above have the following coatings (molar percentages): A, 100% PEG10-OH; B, 10% PEG10-COOH 90% PEG10-OH; C, 25% PEG10-COOH 75% PEG10-OH; D, 50% PEG10-COOH 50% PEG10-OH. (DOCX) [file pone.0109475.s005.docx]

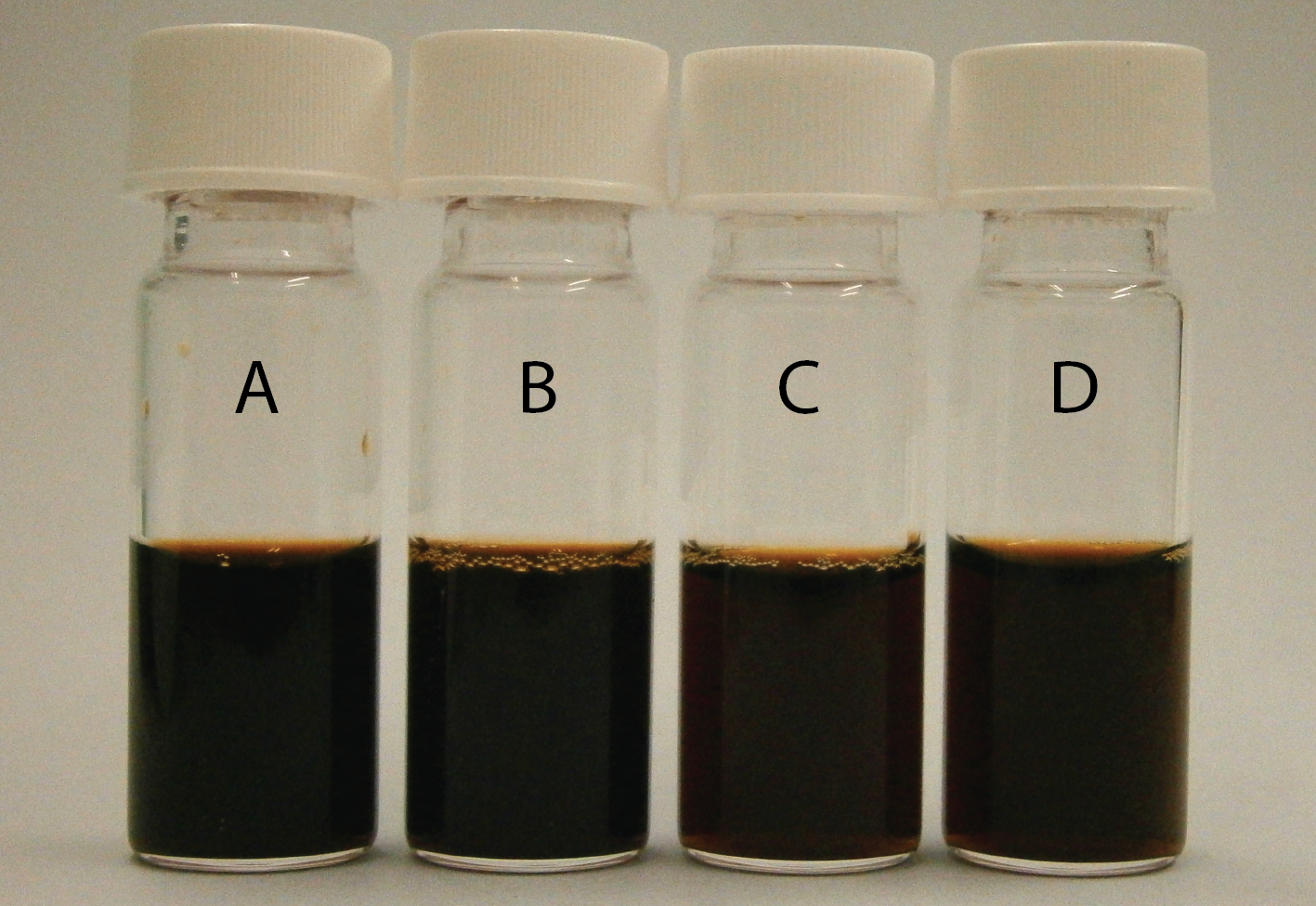


**Figure S5:** **The colloidal stability of the nanoparticle dispersions is excellent, even after 1 year of storage.** The samples (5mg/mL in water, pH 7) show above have the following coatings (molar percentages): **A**, 100% PEG_10_-OH; **B**, 10% PEG_10_-COOH 90% PEG_10_-OH; **C**, 25% PEG_10_-COOH 75% PEG_10_-OH; **D**, 50% PEG_10_-COOH 50% PEG_10_-OH
